# Choque Cardiogênico Devido à Síndrome de Takotsubo de Padrão Invertido Causada por Feocromocitoma: Relato de Caso e Revisão da Literatura

**DOI:** 10.36660/abc.20250558

**Published:** 2026-03-03

**Authors:** Pedro Ivo De Marqui Moraes, Michell Fayad André Haddad, Daniel Bisi de Bôrtoli Valle, Luiz Otávio de Oliveira, Mayara da Silva Custódio, Dirceu Rodrigues de Almeida

**Affiliations:** 1 Universidade Federal de São Paulo São Paulo SP Brasil Universidade Federal de São Paulo, São Paulo, SP – Brasil

**Keywords:** Cardiomiopatia de Takotsubo, Feocromocitoma, Choque cardiogênico

## Introdução

A síndrome de Takotsubo (STT) é caracterizada por um comprometimento agudo da contratilidade miocárdica causado por hiperestimulação simpática após estresse emocional ou físico.^1 , 2^ O balonamento apical é o padrão típico e mais comum, característica que deu origem ao termo clássico descrito por Sato et al. em 1990,^3^ devido à sua semelhança com a histórica armadilha japonesa para pesca de polvos. Padrões atípicos incluem anormalidades de contratilidade da parede médio-basal, basal e focal.

Dados de registros multicêntricos revelam que de 1 a 3% dos pacientes com suspeita de infarto agudo do miocárdio (IAM) recebem, alternativamente, o diagnóstico de STT,^1 , 4^ e as taxas de mortalidade hospitalar são comparáveis às de pacientes com IAM, variando de 3,2 a 10,6%,^5 - 8^ causadas principalmente por choque cardiogênico ou morte súbita por arritmias ventriculares. Ao contrário de outras cardiomiopatias, o déficit de contratilidade ventricular na STT é transitório e a recuperação completa geralmente ocorre em dias ou semanas.^9^

O feocromocitoma é um tumor neuroendócrino raro, secretor de catecolaminas, originário das células cromafins da medula adrenal ou dos paragânglios extra-adrenais.^10^ As manifestações clínicas incluem a tríade clássica de cefaleia, sudorese e palpitações, bem como hipertensão sustentada ou paroxística, hipotensão ortostática, tremores, dor abdominal, perda de peso e ansiedade.^11^ Recentemente, o feocromocitoma tem sido reconhecido como um possível desencadeador da STT.^1 , 2^

## Relato de Caso

Um homem de 65 anos se apresentou com dor torácica intensa e contínua, irradiando para o braço esquerdo, com duração de 2 horas, associada a sudorese e náuseas. A dor iniciou-se durante a atividade sexual após a ingestão de 50 mg de sildenafil. Ele tinha histórico médico de hipertensão, diabetes, hipotireoidismo e um IAM prévio, ocorrido há quatro anos, que não havia sido investigado. Os medicamentos de uso contínuo eram losartana 100 mg/dia, metformina 2.000 mg/dia e levotiroxina 75 mcg/dia. Ele parou de consumir álcool e fumar há 20 anos.

**Figura 1:**
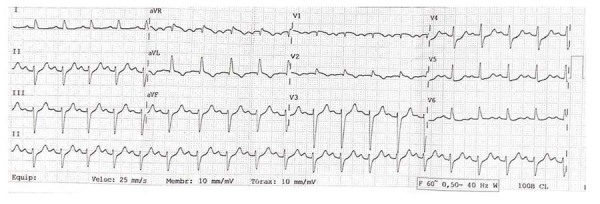
– ECG de admissão: Ritmo sinusal, FC 125 bpm, bloqueio atrioventricular de primeiro grau (intervalo PR de 240 ms), bloqueio fascicular anterior esquerdo, elevação do segmento ST nas derivações aVR, V1 e V2 de até 1,5 mm no ponto J, depressão do segmento ST nas derivações V4 a V6 e intervalo QT corrigido de 434 ms (fórmula de Fridericia).

Ao exame físico na admissão, apresentava diaforese, dispneia, extremidades frias e úmidas, pressão arterial de 70 x 40 mmHg em ambos os membros superiores, frequência cardíaca de 125 bpm, saturação de oxigênio de 85%, murmúrio vesicular bilateral com estertores crepitantes até os ápices, ausculta cardíaca sem sopros, fígado palpável na margem costal direita, ausência de déficits neurológicos focais e peso de 78 kg e altura de 1,69 m. O eletrocardiograma ( Figura 1 ) mostrou ritmo sinusal, frequência cardíaca de 125 bpm, bloqueio atrioventricular de primeiro grau (intervalo PR de 240 ms), bloqueio fascicular anterior esquerdo, supradesnivelamento do segmento ST nas derivações aVR, V1 e V2 de até 1,5 mm no ponto J, infradesnivelamento do segmento ST nas derivações V4 a V6 e intervalo QT corrigido de 434 ms (fórmula de Fridericia).

**Figura 2:**
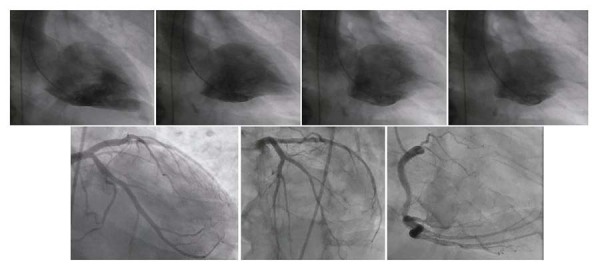
– Cateterismo cardíaco: A angiografia coronária não revelou obstruções significativas das artérias coronárias, e a ventriculografia mostrou hipocinesia médio-basal, hipercinesia apical, válvula mitral sem sinais angiográficos de insuficiência e nenhuma obstrução à ejeção do ventrículo esquerdo.

Diante do diagnóstico de síndrome coronariana aguda e choque cardiogênico, foram prescritos ao paciente aspirina 300 mg por via oral, heparina não fracionada 5.000 UI e furosemida 80 mg por via intravenosa. O paciente necessitou de intubação e norepinefrina 0,35 mcg/kg/min para manter uma pressão arterial média de 60 a 65 mmHg. O cateterismo cardíaco de emergência ( Figura 2 ) não revelou obstruções significativas das artérias coronárias, e a ventriculografia mostrou hipocinesia médio-basal, hipercinesia apical, válvula mitral sem sinais angiográficos de insuficiência e ausência de obstruções à ejeção do ventrículo esquerdo.

Ele evoluiu com índice cardíaco reduzido e disfunção orgânica progressiva, hiperlactatemia, elevação das transaminases, oligúria e disfunção renal, necessitando de hemodiálise. A troponina de alta sensibilidade na admissão foi de 2.308 pg/mL (referência < 14 pg/mL), e um ecocardiograma transtorácico mostrou fração de ejeção do ventrículo esquerdo (FEVE) de 32%, com acinesia médio-basal e hipercinesia apical. Uma tentativa de suporte inotrópico com dobutamina não obteve sucesso, pois houve piora da taquicardia e instabilidade hemodinâmica. O suporte mecânico com balão intra-aórtico por 72 horas permitiu a recuperação dos parâmetros de perfusão e a estabilização hemodinâmica.

**Figura 3:**
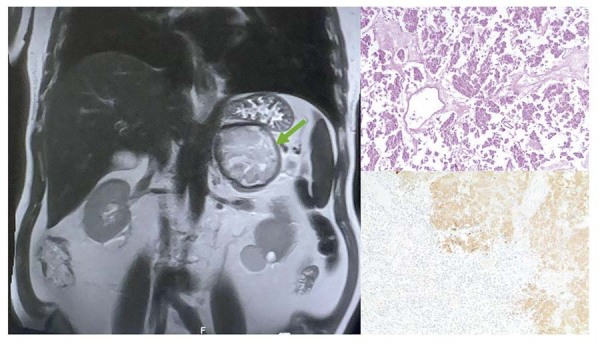
– Ressonância magnética abdominal e exame anatomopatológico pós-adrenalectomia: Massa hemorrágica heterogênea na glândula adrenal esquerda (seta verde), com áreas intralesionais de realce pelo contraste e diâmetro de 88 mm. O exame anatomopatológico confirmou feocromocitoma extensamente necrótico com grau histológico indefinido (PASS) devido a extenso infarto hemorrágico subagudo, com análise imuno-histoquímica positiva para os marcadores neuroendócrinos cromogranina A, sinaptofisina, S100 e enolase.

Durante a investigação de íleo paralítico após a primeira semana na unidade de terapia intensiva (UTI), uma tomografia computadorizada abdominal revelou uma lesão expansiva heterogênea na glândula adrenal esquerda (atenuação de 30 HU e diâmetro de 7,5 x 8,3 cm). Diante dos achados, feocromocitoma e cardiomiopatia adrenérgica induzida por catecolaminas (STT com padrão invertido) se tornaram então as hipóteses diagnósticas preponderantes. O perfil hormonal adrenal mostrou normetanefrina elevada (2,7 nmol/L; referência < 0,9 nmol/L) e cortisol elevado (26,1 mcg/dL; referência < 19,5 mcg/dL), e uma ressonância magnética abdominal ( Figura 3 ) confirmou uma massa hemorrágica heterogênea na glândula adrenal esquerda, com áreas intralesionais de realce pelo contraste e um diâmetro de 88 mm. Devido a limitações institucionais, não foi realizada ressonância magnética cardíaca. Após preparação com bloqueio adrenérgico progressivo e individualizado durante três semanas, até atingir o bloqueio alfa com doxazosina 6 mg duas vezes ao dia, seguido de bloqueio beta com carvedilol 25 mg três vezes ao dia, foi realizada uma adrenalectomia laparoscópica videoassistida sem complicações.

O exame anatomopatológico da peça cirúrgica confirmou feocromocitoma extensamente necrótico com grau histológico (PASS) indefinido devido a extenso infarto hemorrágico subagudo, com análise imuno-histoquímica positiva para os marcadores neuroendócrinos cromogranina A, sinaptofisina, S100 e enolase ( Figura 3 ). Após 60 dias de internação, dos quais 12 em ventilação mecânica e os primeiros 20 na UTI, o paciente recebeu alta no quinto dia pós-operatório com recuperação completa da função orgânica, incluindo FEVE de 74% sem disfunção contrátil segmentar e função renal normal. O paciente realiza acompanhamento regular após 12 meses, assintomático, com tomografia adrenal de controle e níveis de catecolaminas normais.

## Revisão da literatura

A incidência da STT tem aumentado nas últimas décadas, um fato relacionado à exposição a estressores modernos, à maior conscientização da síndrome pela comunidade médica e à expansão das estratégias diagnósticas.^1^ Os resultados do Registro Internacional de Takotsubo (InterTAK), que abrange 3.957 pacientes diagnosticados com STT entre 2004 e 2021 em mais de 40 centros em todo o mundo, destacam tendências temporais como um aumento significativo na proporção de pacientes do sexo masculino (de 10% para 15%), no padrão de déficit contrátil medioventricular (de 18% para 28%) e na prevalência de gatilhos físicos (de 39% para 58%).^5^

Os mecanismos fisiopatológicos da STT não estão completamente elucidados, mas a hiperativação simpática pode desencadear insultos agudos multifacetados, como disfunção microvascular e redução da reserva de fluxo coronário, vasoespasmo macro e microvascular, atordoamento dos cardiomiócitos por efeito catecolaminérgico direto, ativação da cascata inflamatória e desacoplamento energético intracelular.^12^

Os gatilhos emocionais incluem discussões interpessoais ou familiares, mortes inesperadas, desastres naturais e acidentes com múltiplas vítimas, ou mesmo eventos felizes (“Síndrome do Coração Feliz”), como ganhar na loteria ou receber uma festa surpresa.^1 , 6 , 13^Os gatilhos físicos são comuns na prática clínica e incluem doenças do sistema nervoso central (sangramento, hipertensão intracraniana ou trauma), infecções, complicações neoplásicas, quimioterapia, cirurgias e fraturas.^1 , 2 , 5^

O diagnóstico da STT pode ser desafiador, principalmente devido à sua semelhança com o IAM. As alterações eletrocardiográficas comuns incluem elevação do segmento ST, inversão da onda T e prolongamento do intervalo QT, com risco de Torsades de Pointes.^14 , 15^ A elevação da troponina de alta sensibilidade é moderada a alta, mas geralmente não é tão significativa em proporção ao déficit de contratilidade miocárdica, ao contrário do que é tipicamente observado no IAM.^4 , 9^ Em contrapartida, observa-se um aumento substancial nos níveis plasmáticos do peptídeo natriurético tipo B (BNP) e do NT-proBNP, que parece estar diretamente relacionado ao grau de hiperativação simpática.^9^

Não existe uma ferramenta não invasiva estabelecida para o diagnóstico precoce e preciso da STT, sendo a angiografia coronária com ventriculografia considerada o exame padrão-ouro para descartar IAM e confirmar a STT.^1 , 2^ Muitos critérios diagnósticos foram propostos, incluindo os critérios da Clínica Mayo, de Gotemburgo, da Rede Italiana de Takotsubo e os critérios de Madias, entre outros. Recentemente, para aprimorar a identificação e estratificação da STT, foram propostos os critérios InterTAK ( Tabela 1 ). Esses critérios descrevem a justificativa para inovações, como a inclusão do feocromocitoma como possível fator desencadeante e a possibilidade de doença arterial coronariana obstrutiva associada, encontrada em 10 a 29% dos casos, e, portanto, não deve ser considerada um critério de exclusão.^1^

**Tabela 1:** – Critérios diagnósticos InterTAK para a síndrome de Takotsubo

1. Disfunção transitória do ventrículo esquerdo (hipocinesia, acinesia ou discinesia), manifestando-se como balonamento apical ou déficit de contratilidade médio-basal, basal ou focal. Pode haver envolvimento do ventrículo direito. A anormalidade da motilidade geralmente se estende além de uma única distribuição vascular epicárdica.
2. Presença de um gatilho físico, emocional ou combinado (não obrigatório)
3. Lesões neurológicas agudas (hemorragia subaracnoide, acidente vascular cerebral ou convulsões), assim como feocromocitoma, podem servir como gatilhos para a síndrome de Takotsubo.
4. Novas alterações no ECG (elevação do segmento ST, depressão do segmento ST, inversão da onda T e prolongamento do intervalo QTc)
5. Elevação moderada a alta de biomarcadores (troponina). Elevação significativa do peptídeo natriurético tipo B é comum.
6. A doença arterial coronariana obstrutiva não é um critério de exclusão.
7. Os pacientes não apresentam evidências de miocardite infecciosa.
8. As mulheres na pós-menopausa são as mais afetadas.

A ressonância magnética cardíaca é útil na fase subaguda, pois permite a quantificação da função biventricular e a caracterização tecidual por meio da investigação de edema, inflamação e fibrose. Na maioria dos pacientes com STT, o edema miocárdico está presente em regiões com função sistólica anormal, presumivelmente causada por inflamação local.^16 , 17^ O realce tardio de gadolínio geralmente está ausente, mas um grau sutil de fibrose pode estar presente e é um sinal de pior prognóstico.^9 , 18^ O realce tardio também permite a distinção entre STT e outras condições, como IAM (realce subendocárdico ou transmural correspondente ao território vascular) e miocardite (realce mesocárdico, subepicárdico ou irregular).^18^

Pacientes com STT e choque cardiogênico, particularmente aqueles com balonamento apical, devem ser avaliados quanto à obstrução dinâmica da via de saída do ventrículo esquerdo (VSVE), que ocorre em 15 a 20% dos casos.^19 , 20^ O gradiente de pressão dinâmico pode ser avaliado durante o cateterismo cardíaco por meio de manometria de retração com cateter pigtail ou de forma não invasiva por ecocardiografia. Betabloqueadores podem melhorar a obstrução da VSVE, mas são contraindicados em casos de insuficiência cardíaca aguda, hipotensão ou bradicardia.^21^ Como a STT com padrão invertido envolve hipercinesia dos segmentos apicais, o desenvolvimento de obstrução da VSVE é improvável.

Uma revisão sistemática avaliou 104 pacientes com STT induzida por feocromocitoma e comparou os grupos com padrões típicos (balonamento apical, n = 67) versus atípicos (basal ou médio-basal, n = 37).^2 2^ O grupo com padrão atípico apresentou sobrecarga hídrica, insuficiência respiratória e choque cardiogênico significativamente maiores, mas a mortalidade geral foi de 4,8%, sem diferença entre os grupos. Como a taxa estimada de STT desencadeada por feocromocitoma é inferior a 5 a 10%,^1 , 2^ a solicitação de tomografia computadorizada abdominal deve ser motivada por suspeita clínica e não realizada rotineiramente.

O tratamento farmacológico com inibidores da enzima conversora de angiotensina (IECA) ou bloqueadores dos receptores de angiotensina (BRA) tem sido associado à melhora da sobrevida em um ano de acompanhamento em pacientes com STT.^4^ As diretrizes recomendam o uso de IECA ou BRA em combinação com betabloqueadores após a estabilização clínica inicial, pelo menos até a recuperação da função ventricular.^1 , 9^A manutenção da terapia farmacológica a longo prazo deve ser individualizada. A taxa de recorrência da STT é de 4% a 10% nos primeiros 5 anos de acompanhamento, e uma metanálise recente indica que o uso de betabloqueadores ou IECA/BRA não reduziu essa taxa de recorrência.^23^

## Conclusão

A associação entre feocromocitoma e síndrome de Takotsubo reforça a fisiopatologia da hiperativação catecolaminérgica. Reconhecer padrões de disfunção ventricular, particularmente as formas atípicas da síndrome de Takotsubo, que podem estar associadas a piores desfechos clínicos, é importante para o diagnóstico diferencial com infarto agudo do miocárdio e para o tratamento precoce e direcionado.

## References

[1] Singh T, Khan H, Gamble DT, Scally C, Newby DE, Dawson D (2022). Takotsubo Syndrome: Pathophysiology, Emerging Concepts, and Clinical Implications. Circulation.

[2] Ghadri JR, Wittstein IS, Prasad A, Sharkey S, Dote K, Akashi YJ (2018). International Expert Consensus Document on Takotsubo Syndrome (Part I): Clinical Characteristics, Diagnostic Criteria, and Pathophysiology. Eur Heart J.

[3] Sato TH, Uchida T, Dote KMI, Kodama K, Haze K, Hori M (1990). Clinical Aspect of Myocardial Injury: From Ischemia to Heart Failure.

[4] Templin C, Ghadri JR, Diekmann J, Napp LC, Bataiosu DR, Jaguszewski M (2015). Clinical Features and Outcomes of Takotsubo (Stress) Cardiomyopathy. N Engl J Med.

[5] Schweiger V, Cammann VL, Crisci G, Gilhofer T, Schlenker R, Niederseer D (2024). Temporal Trends in Takotsubo Syndrome: Results from the International Takotsubo Registry. J Am Coll Cardiol.

[6] Pätz T, Santoro F, Cetera R, Ragnatela I, El-Battrawy I, Mezger M (2023). Trigger-Associated Clinical Implications and Outcomes in Takotsubo Syndrome: Results from the Multicenter GEIST Registry. J Am Heart Assoc.

[7] Redfors B, Vedad R, Angerås O, Råmunddal T, Petursson P, Haraldsson Iet (2015). Mortality in Takotsubo Syndrome is Similar to Mortality in Myocardial Infarction - A Report from the SWEDEHEART Registry. Int J Cardiol.

[8] Almeida GLG, Mansur J, Albuquerque DC, Xavier SS, Pontes Á, Gouvêa EP (2020). Takotsubo Multicenter Registry (REMUTA) - Clinical Aspects, In-Hospital Outcomes, and Long-Term Mortality. Arq Bras Cardiol.

[9] Ghadri JR, Wittstein IS, Prasad A, Sharkey S, Dote K, Akashi YJ (2018). International Expert Consensus Document on Takotsubo Syndrome (Part II): Diagnostic Workup, Outcome, and Management. Eur Heart J.

[10] Neumann HPH, Young WF, Eng C (2019). Pheochromocytoma and Paraganglioma. N Engl J Med.

[11] Saavedra T JS, Nati-Castillo HA, Cometa LAV, Rivera-Martínez WA, Asprilla J, Castaño-Giraldo CM (2024). Pheochromocytoma: An Updated Scoping Review from Clinical Presentation to Management and Treatment. Front Endocrinol.

[12] Lyon AR, Citro R, Schneider B, Morel O, Ghadri JR, Templin C (2021). Pathophysiology of Takotsubo Syndrome: JACC State-of-the-Art Review. J Am Coll Cardiol.

[13] Stiermaier T, Walliser A, El-Battrawy I, Pätz T, Mezger M, Rawish E (2022). Happy Heart Syndrome: Frequency, Characteristics, and Outcome of Takotsubo Syndrome Triggered by Positive Life Events. JACC Heart Fail.

[14] Namgung J (2014). Electrocardiographic Findings in Takotsubo Cardiomyopathy: ECG Evolution and Its Difference from the ECG of Acute Coronary Syndrome. Clin Med Insights Cardiol.

[15] Chhabra L, Butt N, Ahmad SA, Kayani WT, Sangong A, Patel V (2021). Electrocardiographic Changes in Takotsubo Cardiomyopathy. J Electrocardiol.

[16] Migliore F, Zorzi A, Marra MP, Iliceto S, Corrado D (2015). Myocardial Edema as a Substrate of Electrocardiographic Abnormalities and Life-Threatening Arrhythmias in Reversible Ventricular Dysfunction of Takotsubo Cardiomyopathy: Imaging Evidence, Presumed Mechanisms, and Implications for Therapy. Heart Rhythm.

[17] Neil C, Nguyen TH, Kucia A, Crouch B, Sverdlov A, Chirkov Y (2012). Slowly Resolving Global Myocardial Inflammation/Oedema in Tako-Tsubo Cardiomyopathy: Evidence from T2-Weighted Cardiac MRI. Heart.

[18] Eitel I, von Knobelsdorff-Brenkenhoff F, Bernhardt P, Carbone I, Muellerleile K, Aldrovandi A (2011). Clinical Characteristics and Cardiovascular Magnetic Resonance Findings in Stress (Takotsubo) Cardiomyopathy. JAMA.

[19] Liu K, Sun Z, Wei T (2015). "Reverse McConnell's Sign": Interpreting Interventricular Hemodynamic Dependency and Guiding the Management of Acute Heart Failure during Takotsubo Cardiomyopathy. Clin Med Insights Cardiol.

[20] Lousinha A, Gilkeson R, Bezerra H (2012). Left Ventricular Outflow Tract Obstruction and Takotsubo Syndrome. Rev Port Cardiol.

[21] Madias JE (2016). If Channel Blocker Ivabradine vs. ß-blockers for Sinus Tachycardia in Patients with Takotsubo Syndrome. Int J Cardiol.

[22] Aw A, de Jong MC, Varghese S, Lee J, Foo R, Parameswaran R (2023). A Systematic Cohort Review of Pheochromocytoma-Induced Typical versus Atypical Takotsubo Cardiomyopathy. Int J Cardiol.

[23] Santoro F, Sharkey S, Citro R, Miura T, Arcari L, Urbano-Moral JA (2024). Beta-Blockers and Renin-Angiotensin System Inhibitors for Takotsubo Syndrome Recurrence: A Network Meta-Analysis. Heart.

