# Supplementary material for: Choque Cardiogênico Devido à Síndrome de Takotsubo de Padrão Invertido Causada por Feocromocitoma: Relato de Caso e Revisão da Literatura
Source: Arq Bras Cardiol. 2026 Mar 3;123(2):e20250558. [Article in Portuguese] doi: 10.36660/abc.20250558 (PMC13128190; doi:10.36660/abc.20250558)
Supplement: Translation

# Translation: Cardiogenic Shock Due to Inverted-Pattern Takotsubo Syndrome Caused by Pheochromocytoma: Case Report and Literature Review

**DOI:** 10.36660/abc.20250558i

**Authors:** Pedro Ivo De Marqui Moraes, Michell Fayad André Haddad, Daniel Bisi de Bôrtoli Valle, Luiz Otávio de Oliveira, Mayara da Silva Custódio, Dirceu Rodrigues de Almeida

**Affiliations:** 1 Brazil Universidade Federal de São Paulo, São Paulo, SP – Brazil

**Keywords:** Takotsubo Cardiomyopathy, Pheochromocytoma, Cardiogenic Shock

## Introduction

Takotsubo syndrome (TTS) is characterized by an acute impairment of myocardial contractility caused by sympathetic hyperstimulation after emotional or physical stress.^1 , 2^Apical ballooning is the typical and most common pattern, a feature that gave rise to the classic term described by Sato et al. in 1990,^3^due to its similarity to the historical Japanese octopus trap. Atypical patterns include mid-basal, basal, and focal wall motion abnormalities.

Data from multicenter registries reveal that 1 to 3% of patients suspected of having an acute myocardial infarction (AMI) are alternatively diagnosed with TTS,^1 , 4^and in-hospital mortality rates are comparable to those of patients with AMI and range from 3.2 to 10.6%,^5 - 8^mainly caused by cardiogenic shock or sudden death from ventricular arrhythmias. In contrast to other cardiomyopathies, the ventricular contractility deficit in TTS is transient, and complete recovery usually occurs within days to weeks.^9^

Pheochromocytoma is a rare catecholamine-secreting neuroendocrine tumor originating from chromaffin cells in the adrenal medulla or extra-adrenal paraganglia.^10^Clinical manifestations include the classic triad of headache, sweating, and palpitations, as well as sustained or paroxysmal hypertension, orthostatic hypotension, tremors, abdominal pain, weight loss, and anxiety.^11^Recently, pheochromocytoma has been recognized as a possible trigger for TTS.^1 , 2^

## Case Report

A 65-year-old male presented with severe and continuous chest pain, radiating to the left arm lasting 2 hours, associated with sweating and nausea. This pain began during sexual activity after the intake of sildenafil 50 mg. He had a medical history of hypertension, diabetes, hypothyroidism, and a previous AMI four years ago that had not been investigated. Continuous use medications were losartan 100 mg/day, metformin 2,000 mg/day, and levothyroxine 75 mcg/day. He stopped drinking alcohol and smoking 20 years ago.

His physical examination upon admission exhibited diaphoresis, dyspnea, cold and clammy extremities, blood pressure 70 x 40 mmHg in both upper limbs, heart rate of 125 bpm, oximetry of 85%, ventricular murmurs present bilaterally with crackling rales up to the apices, normal heart sounds without murmurs, palpable liver at the right costal margin, absence of focal neurological deficits and a referred weight of 78 kg and height 1.69 m. The electrocardiogram ( Figure 1 ) showed sinus rhythm, heart rate 125 bpm, first-degree atrioventricular block (PR interval 240 ms), left anterior fascicular block, ST-segment elevation in leads aVR, V1, and V2 up to 1.5 mm at the J point, ST-segment depression in leads V4 to V6, and a corrected QT interval of 434 ms (Fridericia formula).

**Figure 1:** – Admission ECG: Sinus rhythm, HR 125 bpm, first-degree atrioventricular block (PR interval 240 ms), left anterior fascicular block, ST-segment elevation in leads aVR, V1, and V2 up to 1.5 mm at the J point, ST-segment depression in leads V4 to V6, and a corrected QT interval of 434 ms (Fridericia formula).

Given the diagnosis of acute coronary syndrome and cardiogenic shock, he was prescribed aspirin 300 mg orally, unfractionated heparin 5,000 IU, and furosemide 80 mg intravenously. The patient required intubation and norepinephrine 0.35 mcg/kg/min to maintain a mean arterial pressure of 60 to 65 mmHg. Emergency cardiac catheterization ( Figure 2 ) revealed no significant coronary artery obstructions, and ventriculography showed mid-basal hypokinesia, apical hyperkinesia, a mitral valve without angiographic signs of insufficiency, and no obstructions to left ventricular ejection.

**Figure 2:** – Cardiac catheterization: Coronary angiography revealed no significant coronary artery obstructions, and ventriculography showed mid-basal hypokinesia, apical hyperkinesia, a mitral valve without angiographic signs of insufficiency, and no obstructions to left ventricular ejection

He evolved with reduced cardiac index and progressive organ dysfunction, hyperlactatemia, elevated transaminases, oliguria, and renal dysfunction requiring hemodialysis. Admission high-sensitivity troponin was 2,308 pg/mL (reference < 14 pg/mL), and a transthoracic echocardiogram showed a left ventricular ejection fraction (LVEF) of 32%, with mid-basal akinesia and apical hyperkinesia. An attempt at inotropic support with dobutamine was unsuccessful, as there was a worsening of the tachycardia and hemodynamic instability. Mechanical support with an intra-aortic balloon pump for 72 hours allowed recovery of perfusion parameters and hemodynamic stabilization.

During the investigation of paralytic ileus after the first week in the intensive care unit (ICU), an abdominal computed tomography scan revealed a heterogeneous expansile lesion in the left adrenal gland (attenuation of 30 HU and diameter of 7.5 x 8.3 cm). Pheochromocytoma and catecholamine-induced adrenergic cardiomyopathy (Inverted-pattern TTS) then became the preponderant diagnostic hypothesis. The adrenal hormone profile showed elevated normetanephrine 2.7 nmol/L (reference < 0.9 nmol/L) and cortisol 26.1 mcg/dL (reference < 19.5 mcg/dL), and an abdominal magnetic resonance imaging ( Figure 3 ) confirmed a heterogeneous hemorrhagic mass in the left adrenal gland, with intralesional areas of contrast enhancement and a diameter of 88 mm. In the face of institutional constraints, a cardiac magnetic resonance imaging was not performed. After preparation with progressive and individualized adrenergic blockade for three weeks until achieving alpha-blockade with doxazosin 6 mg twice daily, followed by beta-blockade with carvedilol 25 mg three times daily, a video-assisted laparoscopic adrenalectomy was performed without complications.

**Figure 3:** – Abdominal magnetic resonance imaging and post-adrenalectomy pathology: Hemorrhagic heterogeneous mass in the left adrenal gland (green arrow), with intralesional areas of contrast enhancement and a diameter of 88 mm. Pathology confirmed extensively necrotic pheochromocytoma with an unavailable histological grade (PASS) due to extensive subacute hemorrhagic infarction, with positive immunohistochemical analysis for the neuroendocrine markers chromogranin A, synaptophysin, S100, and enolase.

Pathology examination of the surgical specimen confirmed extensively necrotic pheochromocytoma with an unavailable histological grade (PASS) due to extensive subacute hemorrhagic infarction, with positive immunohistochemical analysis for the neuroendocrine markers chromogranin A, synaptophysin, S100, and enolase ( Figure 3 ). After 60 days of hospitalization, of which 12 days were on mechanical ventilation and the first 20 in the ICU, the patient was discharged on the fifth postoperative day with complete recovery of organ function, including a LVEF of 74% without segmental contractility dysfunction and normal kidney function. The patient undergoes regular follow-up after 12 months, asymptomatic, with normal control adrenal tomography and catecholamine levels.

## Literature Review

The incidence of TTS has been increasing in recent decades, a fact related to exposure to modern-day stressors, increased awareness of the syndrome by the medical community, and expanded diagnostic strategies.^1^Results from the International Takotsubo Registry (InterTAK), covering 3,957 patients diagnosed with TTS between 2004 and 2021 in more than 40 centers worldwide, highlight temporal trends as a significant increase in the proportion of male patients (from 10% to 15%), the midventricular contractile deficit pattern (from 18% to 28%) and the prevalence of physical triggers (from 39% to 58%).^5^

The pathophysiological mechanisms of TTS are not completely elucidated, but sympathetic hyperactivation can trigger multifaceted acute insults such as microvascular dysfunction and reduced coronary flow reserve, macro and microvascular vasospasm, cardiomyocyte stunning by direct catecholaminergic effect, activation of the inflammatory cascade, and intracellular energy uncoupling.^12^

Emotional triggers include interpersonal or family arguments, unexpected deaths, natural disasters, and accidents with multiple victims, or even happy events (“Happy Heart Syndrome”) such as winning a lottery or receiving a surprise party.^1 , 6 , 13^Physical triggers are common in clinical practice and include central nervous system diseases (bleeding, intracranial hypertension, or trauma), infections, neoplastic complications, chemotherapy, surgeries, and fractures.^1 , 2 , 5^

Diagnosing TTS can be challenging, particularly due to its similarity to AMI. Common electrocardiographic changes are ST-segment elevation, T-wave inversion, and QT interval prolongation with a risk of Torsade de Pointes.^14 , 15^High-sensitivity troponin elevation is moderate to high, but it is usually not as significant in proportion to the myocardial contractility deficit, unlike what is typically seen in AMI.^4 , 9^In contrast, a substantial increase in the plasma levels of B-type natriuretic peptide (BNP) and NT-proBNP is seen and appears to be directly related to the degree of sympathetic overactivation.^9^

There is no established non-invasive tool for early and accurate diagnosis of TTS, and coronary angiography with ventriculography is considered the gold standard diagnostic test for ruling out AMI and confirming TTS.^1 , 2^Many diagnostic criteria have been proposed, including the Mayo Clinic, Gothenburg, Takotsubo Italian Network Proposal, Madias criteria, among others. Recently, to improve the identification and stratification of TTS, the InterTAK criteria ( Table 1 ) were proposed. These criteria outline the rationale for innovations, such as the inclusion of pheochromocytoma as a possible trigger and the possibility of associated obstructive coronary artery disease, found in 10 to 29% of cases, and therefore should not be considered an exclusion criterion.^1^

**Table 1:** – InterTAK diagnostic criteria for Takotsubo Syndrome

1.Transient left ventricular dysfunction (hypokinesia, akinesia, or dyskinesia), manifesting as apical ballooning or mid-basal, basal, or focal contractility deficit. Right ventricular involvement may be present. The motility abnormality usually extends beyond a single epicardial vascular distribution.
2.Presence of a physical, emotional, or combined trigger (not mandatory)
3.Acute neurological insults (subarachnoid hemorrhage, stroke, or seizures), as well as pheochromocytoma, can serve as triggers for Takotsubo syndrome.
4.New ECG changes (ST-segment elevation, ST-segment depression, T-wave inversion, and QTc prolongation)
5.Moderate to high elevation of biomarkers (troponin). Significant elevation of B-type natriuretic peptide is common.
6.Obstructive coronary artery disease is not an exclusion criterion.
7.Patients have no evidence of infectious myocarditis.
8.Postmenopausal women are predominantly affected.

Cardiac magnetic resonance imaging is useful in the subacute phase, as it allows quantification of biventricular function and tissue characterization by investigating edema, inflammation, and fibrosis. In most patients with TTS, myocardial edema is present in regions with abnormal systolic function, presumably caused by local inflammation.^16 , 17^Late gadolinium enhancement is usually absent, but a subtle degree of fibrosis may be present and is a sign of a worse prognosis.^9 , 18^Late enhancement also allows the distinction between TTS and other conditions, such as AMI (subendocardial or transmural enhancement corresponding to the vascular territory) and myocarditis (mesocardiac, subepicardial, or patchy enhancement).^18^

Patients with TTS and cardiogenic shock, particularly those with apical ballooning, should be evaluated for dynamic left ventricular outflow tract (LVOT) obstruction, which occurs in 15 to 20% of cases.^19 , 20^The dynamic pressure gradient can be assessed during cardiac catheterization using pigtail catheter retraction manometry or noninvasively using echocardiography. Beta-blockers can improve LVOT obstruction, but are contraindicated in cases of acute heart failure, hypotension, or bradycardia.^21^Since inverted-pattern Takotsubo involves hyperkinesis of apical segments, the development of LVOT obstruction is unlikely.

A systematic review evaluated 104 patients with pheochromocytoma-induced TTS and compared the groups of typical (apical ballooning n = 67) versus atypical (basal or mid-basal n = 37) patterns.^22^The group with the atypical pattern presented significantly greater fluid overload, respiratory failure, and cardiogenic shock, but overall mortality was 4.8%, with no difference between the groups. Since the estimated rate of TTS triggered by pheochromocytoma is less than 5 to 10%,^1 , 2^the request for abdominal CT scans should be motivated by clinical suspicion and not performed routinely.

Pharmacological treatment with angiotensin-converting enzyme inhibitors (ACEIs) or angiotensin receptor blockers (ARBs) has been associated with improved survival at 1-year follow-up in TTS patients.^4^Guidelines recommend the use of ACEIs or ARBs in combination with beta-blockers after initial clinical stabilization, at least until recovery of ventricular function.^1 , 9^Maintenance of long-term pharmacological therapy should be individualized. The recurrence rate of TTS is 4% to 10% in the first 5 years of follow-up, and a recent meta-analysis indicates that the use of beta-blockers or ACEIs/ARBs did not reduce this recurrence rate.^23^

## Conclusion

The association of pheochromocytoma and Takotsubo Syndrome reinforces the pathophysiology of catecholaminergic hyperactivation. Recognizing patterns of ventricular dysfunction, particularly the atypical forms of TTS, which may be associated with worse clinical outcomes, is important for the differential diagnosis with AMI and for early and targeted treatment.
